# Validating Confined Flame Noise Simulation Using External Sensor

**DOI:** 10.3390/s22208039

**Published:** 2022-10-21

**Authors:** Andrew J. Williamson, Shubham Srivastava, Khaled A. Sallam

**Affiliations:** 1 School of Mechanical and Aerospace Engineering, Oklahoma State University, Tulsa, OK 74106, USA; 2 Rheem Water Heating, Montgomery, AL 36109, USA

**Keywords:** combustion, acoustics, furnace, noise, acoustic analogy

## Abstract

Advancements in lean premixed combustion have increased the efficiency and reduced the amount of greenhouse gas emissions, but they have led to increased noise emissions due to higher turbulence and mixing fluctuations. This study used an external sensor (microphone) to validate the simulation of the combustion noise of a confined space. An experimental facility with a laboratory-scale furnace was used to carry out the measurement, and the simulation of the confined flame noise was conducted in OpenFOAM. The simulation utilized the Partially Stirred Reactor (PaSR) and a hybrid computational aeroacoustics (CAA) approach using the large eddy simulation (LES)/the Ffwocs Williams–Hawkings (FWH) method. Additionally, unsteady Reynolds-averaged Navier–Stokes (URANS)/the FWH method was tested for a comparison with the LES prediction. A sensor which was placed outside the enclosure for ease of access was then used to validate the results of the numerical model. The sensor data agreed with the LES/FWH results including the amplitude and frequency of the primary combustion peak and the overall sound pressure level (OASPL). This suggested that a sensor which was placed outside the enclosure could serve as a validation tool for the simulation of the confined flames despite the sound reflections from the walls.

## 1. Introduction

Advanced combustion systems have an increased efficiency and reduce their greenhouse gas emissions, but they have led to an increased noise emission due to the higher turbulence and mixing fluctuations that are associated with lean combustion. Noise pollution can cause serious structural damage if it is not controlled. The literature about combustion noise is dominated by work that is related to jet noise with applications to aircraft and rocket engines. However, the study of combustion noise is also important for confined combustion applications, extending from large-scale furnaces to smaller residential spaces and water heating systems. Commercially, the noise reduction of residential furnaces is done with an iterative design process. This process can be costly and time consuming to employ. With the advancements in computational fluid dynamics (CFD), the iterative design process can be accelerated by the simulation of noise levels for varying design geometries.

Combustion noise has been studied for different configurations, e.g., open and confined flames [1,2], gasoline engines [3], and diesel engines [4,5]. The recent reviews of the computational methods include those that were performed by Zandie et al. [6], Dowling and Mahmoudi [7], Ihme [8], and Tam et al. [9]. Combustion noise is caused by direct and indirect mechanisms [10]. Direct noise is related to the inherent unsteadiness of the turbulent combustion and the heat release fluctuations that are caused by the reactive region, whereas indirect noise is generated due to the acceleration of the flow inhomogeneities (entropy, vorticity, and composition) [11]. Computational Combustion Acoustics (CCA) is a relatively new field, and it uses many tools from CAA and computational flame dynamics fields. There are two main problems in the CCA: (1) determining the source of noise, which requires the modeling of small-scale structures, and (2) the modeling of sound propagation into the far-field, of which the dimensions are generally orders-of-magnitude larger than the combustion region. Since the computational domain sizes are completely different, calculating both problems in the same model is an expensive solution and generally, the two problems are dealt with separately. 

The CAA techniques include direct computations and hybrid computations. The direct approach solves the sound issue by solving the compressible flow equations, and the simulation must be sufficiently large to include the source of the noise and part of the near field. The hybrid approach decouples the sound calculations from the flow calculations. The far-field sound is obtained by the solution of an acoustic analogy using the computed source field data. This method assumes that the flow generates sound, but the sound does not affect the flow significantly. The length scales of the acoustic wavelength are significantly different from the acoustic source [12]. The difference in the scale for the fluid and acoustic disturbances requires a high degree of numerical accuracy in the direct method, but the hybrid approach numerical accuracy in the flow is less critical, allowing for lower resolution schemes [13]. In the confined flows, the sound propagation must be simulated over a large domain. The hybrid schemes generally use LES to calculate the fluctuations, then in the second step, the sound radiation is calculated using perturbed Euler equations. The far-field noise can also be determined by the extrapolation of the nearfield values. Lighthill’s acoustic analogy [14,15,16] is commonly used, however, there are several other analogies, each of which account for different effects. 

The present study explores the validation of an acoustic analogy method for simulating noise in a laboratory-scale furnace using an external sensor. The sensor was conveniently installed outside the furnace due to there being limited access to the combustion chamber.

## 2. Experimental Methods

**Figure 1:**
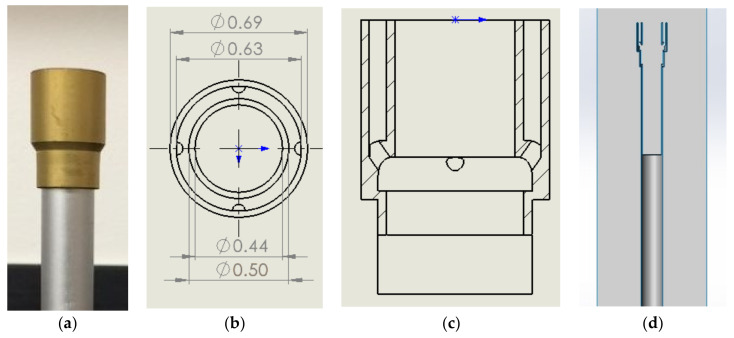
(**a**) Image of a Bunsen burner stabilizer, (**b**) dimensional drawing of the stabilizer from the top, (**c**) section view of the Bunsen burner revealing the small openings in the annulus, and (**d**) the inverted flow domain for the simulation.

**Figure 2:**
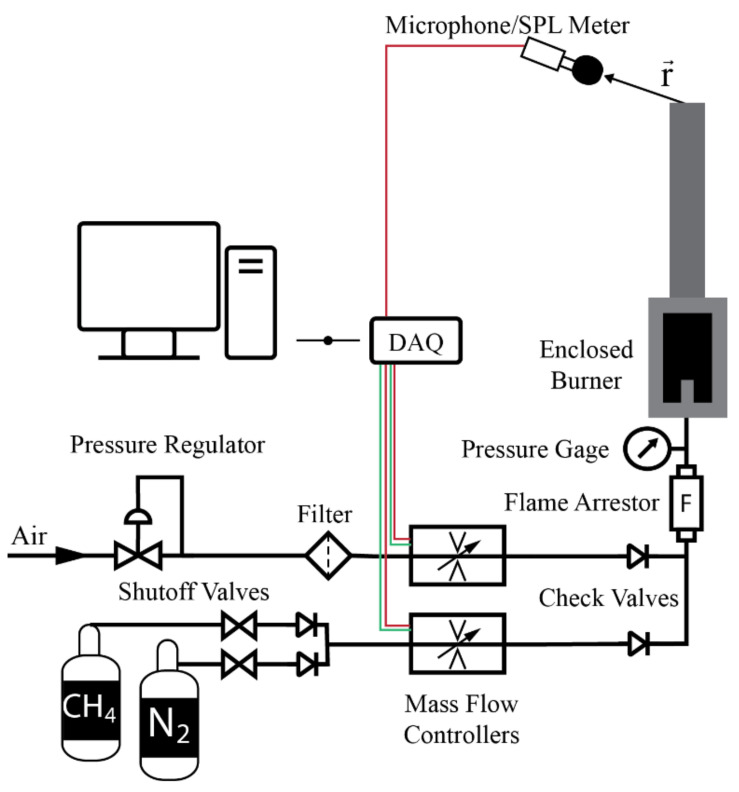
Diagram of the experimental setup.

**Figure 3:**
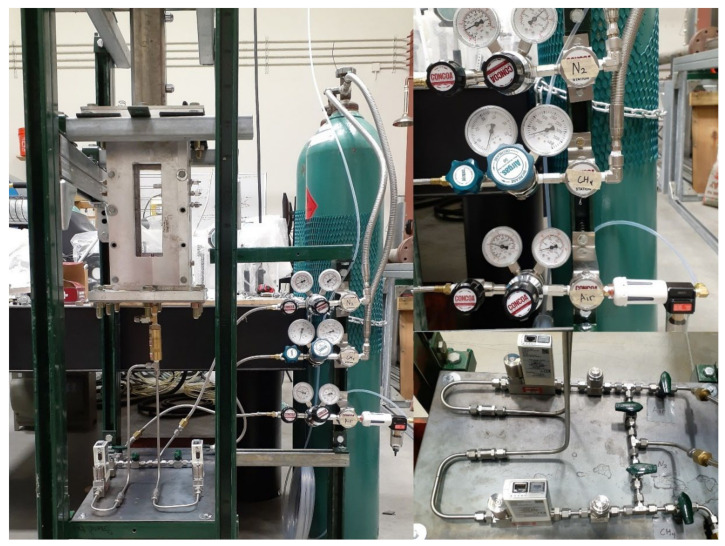
Photographs of the experimental setup.

The experimental setup was designed to deliver premixed CH_4_ and air into a burner (Figure 1) which was placed in confined space. The diagram of the setup is shown in Figure 2, and photographs of the experiment are shown in Figure 3. An external sensor (model UMIK-1 USB measurement-calibrated microphone) and a sound pressure level (SPL) meter (model Larson Davis 831) were placed a distance $\vec{r}=$ (3, 3, 6) inches from the center line of the outlet plane. The placement of the sensor can affect the amplitude of the measurements, particularly at the lower frequencies that are produced by combustion. The microphone must be placed in the far-field for its position which is accurately measured to be modeled with the acoustic analogy. The microphone was connected to the computer, and it simultaneously recorded the sound and measured the SPL spectrum using the REW software [17]. The SPL meter recorded the internal memory, then, the recorded data were transferred to the computer.

Shop air was piped to the setup, and it was filtered for particulates and moisture. N_2_ was used to purge the system post-combustion to remove any combustible gases from the setup and for calibrating the Bronkhorst mass flow controllers: 10 L/min for the air and 1 L/min for the CH_4_. The mass flow controllers were pre-loaded with the capability for measuring the liter per minute of air and CH_4_. The lean mixture was delivered with an air-to-fuel equivalence ratio λ = 1.05 at a flow rate of 7.7 L/min. The burner was placed in a (1.5 × 2.5 × 0.25) inch square pipe that was made from stainless steel 304 with rounded corners. The viewing chamber was aluminum with quartz windows. A digital single reflex lens (DSLR) camera was placed to record video and photos of the flame. The area from the base of the viewing chamber to the outlet of the square pipe was 27.5 inches. The burner outlet was a standard Bunsen burner design with a stabilizer, and it was positioned 7 inches from the bottom of the experimental duct.

## 3. Computational Methods

The CFD simulations were performed using OpenFOAM [18], an object-oriented library that is written in C++ programming language, which was originally developed in 1998. OpenFOAM is an open-source database with a large user base from both academic and commercial organizations. It uses the finite volume method (FVM) for solving the governing equations for CFD. The fork of OpenFOAM v2012 was used, which was developed by OpenCFD Ltd. [19]. The benefit of using OpenFOAM in comparison with the commercial software is the customizability of it and the community within it. With OpenFOAM you can take an existing version and modify it to fit your specific needs and you can take advantage of the rapid advances that are made by the CFD community without having to code a new methodology.

A hybrid CFD and acoustic analogy method was used in this study. The acoustic analogy was implemented using the free open-source library addition to OpenFOAM called libAcoustics, which was developed by Epikhin et al. in 2015 [20]. They validated the method against the experimental results of the rod–airfoil noise interaction using the Curle analogy. The libAcoustics library was developed for far-field noise computations, and it contains predictions using the Curle analogy [21], the FWH analogy [22,23,24], and the CFD/boundary element method coupling. The libAcoustics library contains formulations for the FWH analogy: the Farassat 1A formulation [25] and the Garrick Triangle (GT) formulation [26]. The *libAcoustics* library is not a standard library for OpenFOAM, and it requires a separate installation. The library is available on GitHub for multiple versions of OpenFOAM, and it is currently being maintained by the UniCFD Web-laboratory [27]. In an earlier study [28], the FWH analogy was also validated using another software using the rod–airfoil interaction. The library has been used to simulate compressible free jets, and the results have been compared favorably to the experimental data using the FWH method [29,30]. It has also been used to simulate the marine propeller noise [31] and the rotor noise from unmanned arial vehicles [32,33]. Airfoil-generated noise and jet noise has been successfully simulated utilizing the libAcoustics implementation of the FWH method. Unfortunately, most of the previous studies have focused on the non-reacting flows. Though the library has been tested for the confined flows for the cavity wall noise [34]. 

Hybrid methods for predicting combustion noise have been implemented previously in OpenFOAM [35,36], and others [37,38] have been implemented on open flames. Much of the literature of confined flames involves noise prediction in the swirl combustors [39,40,41,42,43]. The literature is lacking in information about the low Reynolds number confined flow with combustion as well as noise prediction for low Reynolds number flow with non-swirling flame geometries. Validations of the acoustic analogy method using the CFD/FWH method for confined combustion is also lacking. This study aims to validate the applicability of a hybrid CFD/CAA method using the LES/FWH and URANS/FWH methods by modeling a laboratory scale furnace in OpenFOAM.

### 3.1. Burner Geometry and Computation Grid

The standard Bunsen burner stabilizer that is displayed in Figure 1a had a 0.5-inch outer diameter and an approximately a 0.44-inch inner diameter. It has an annular opening around the stabilizer that has an inner diameter of approximately 0.63 inches and an outer diameter of 0.69 inches as demonstrated in Figure 1b. The annular portion of the Bunsen burner had four small holes which were located at equidistant points around the opening as shown in Figure 1c. These openings were estimated based on a visual inspection of the burner stabilizer. The Bunsen burner and pipe system were modeled, then, inverted to the flow domain that is demonstrated in Figure 1d.

**Figure 4:**
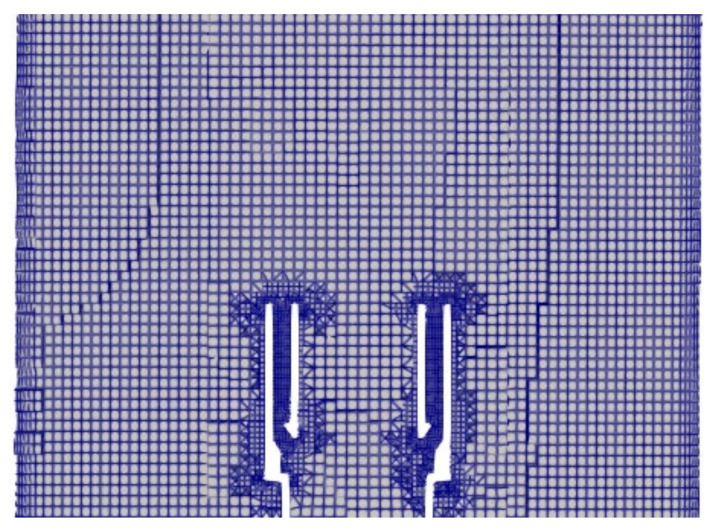
A portion of the mesh surrounding the burner stabilizer.

The mesh that is shown in Figure 4 had a maximum cell size of 2 mm. A mesh refinement region near the burner outlet with 1 mm cells was used to properly resolve of the combustion, wake, and recirculation regions. The cells in the annulus were refined additionally to resolve the geometry and flow in those regions. Boundary layers were added to the pipe exterior due to the thermal boundary conditions. The mesh had approximately 1.7 million cells.

### 3.2. Simulation Settings

The solver that was used for this simulation was reactingFoam, which is a compressible transient flow solver. The simulation settings are listed in Table 1. The first order Euler time scheme was used for the transient simulations. The tolerance was set to 10^−10^ to resolve the small pressure perturbations, but not to slow down the simulation too greatly. The time step was set to 10 μs to ensure stability and to reduce the computational time.

The initial and boundary conditions for the simulation are listed in Table 2. The species inlet mass fraction was set to match the experimental conditions: $\dot{V}=$ 7.7 L/min and λ = 1.05. The temperature on the pipe was set to simulate the convection heat transfer based on the thermal resistance since the temperature of the mean flow could affect the acoustic predictions.

The simulation was performed with turbulence modeling since combustion causes there to be fluctuations in the flow. The k−ϵ closure model was utilized in conjunction with URANS turbulence model. The wall-adapting local eddy-viscosity subgrid-scale model for LES was used since the previous studies have shown good results for a confined reacting flow [44,45]. In both cases, the wall functions were used for turbulent viscosity $\nu_{t}$ and thermal diffusivity $\alpha_{t}$. The y^+^ for the burner interior was consistently less than 5, with there being a maximum of 6.8. The y^+^ for the burner exterior and pipe wall was less than 1 for all of the cells. A compressible wall function was utilized to calculate the thermal diffusivity based on the turbulent viscosity and turbulent Prandtl number.

The turbulent combustion model that was used was the PaSR model with the skeletal reaction mechanism Z42 [46], which was shown previously to be a good compromise between the speed and accuracy [47]. The value of the kinematic viscosity air for high temperatures is in the order of 10^−6^, and the kinetic energy and turbulent dissipation can be estimated from the burner geometry, and with these the mixing constant was estimated, $C_{mix}\approx$ 0.1. 

The *libAcoustics* library was utilized for the virtual microphones using the FWH analogy. The control surface that was selected was the wall of the pipe. The reference properties that were used were for the ambient air conditions since the microphone was not inside the pipe. The GT formulation was used since it reduces the computational cost, and the assumptions for the stationary microphone and acoustic source are valid in this case. The libAcoustics library comes with a built-in SPL-spectrum calculator, however, in this case, a program was developed for the signal processing. The microphone settings were 32,000 Hz and 16-bit measurement. One full second was processed for a resolution of 1 Hz SPL-frequency spectrum with a Hann window and no averaging. The virtual microphone had a sampling frequency of 100,000 samples per second. One half a second of data was processed and normalized to 1 Hz with a Hann window and no averaging. 

## 4. Results and Discussion

### 4.1. Flow Visualization

**Figure 5:**
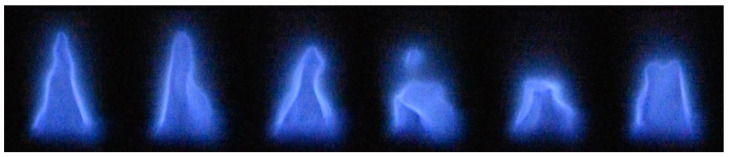
Still images from the video recordings demonstrating the wrinkling of the burner’s flame cone.

The DSLR camera recorded the videos of the flames during the combustion process. Once the flames reached their maximum velocity, the fluctuations and the wrinkling of the flame were observed. The exposure was reduced to capture the flame cone. Still images were taken from the videos to demonstrate the flame wrinkling, and these are placed in Figure 5. The flame wrinkling occurred faster than the camera could capture, so the images do not represent a single period of wrinkling. Additionally, the wrinkling appeared to be unsteady and random rather than periodic in nature like it is expected to be in a tube configuration. The cause of the wrinkling in the flame normally would be attributed to the interaction with the wall and recirculation regions. However, since there was no flow straightener in the device, there was likely influence from the upstream turbulence that was caused by the nozzle being located at the inlet of the burner pipe. This burner inlet was not simulated due to the increased computation cost making it unfeasible with the study’s current resources.

**Figure 6:**
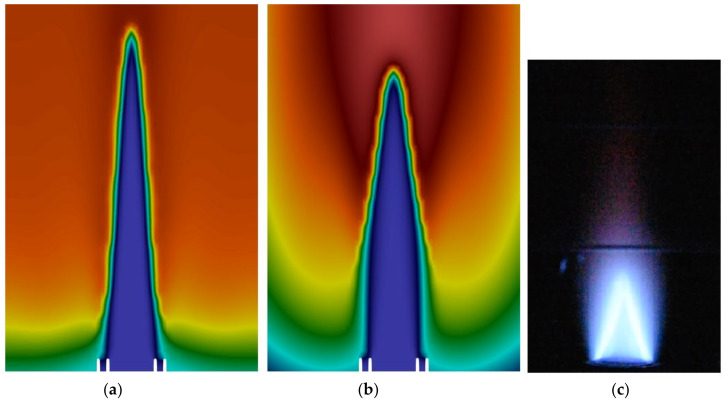
Contour of the flame temperature: (**a**) LES and (**b**) URANS in comparison with (**c**) the experiment.

The flame image was captured with a higher exposure to measure the height of the flame to compare it with the simulation. The flame height was determined by using the diameter of the burner as a reference, then, it was measuring the distance from the center of the burner outlet to the point of reference. The height of the flame cone was measured to be approximately 0.82 inches. The temperature of the flames for the URANS and LES simulations were compared to the flame images in Figure 6. The simulations significantly overestimated the size of the flame cone, showing a non-reacting region that was three times the height of that which was measured in the image. This is an example of the drawbacks of the PaSR method since the $C_{mix}$ parameter must be estimated before the simulation. By reducing the $C_{mix}$ parameter, there would be a reduction in the flame cone height. Additionally, the wrinkling of the flame cone did not occur in the simulation that was observed in the experiment. This indicates that the primary cause was as it was suspected to be from the upstream turbulence in the experiment since the inlet of the burner pipe was not simulated, thus demonstrating the importance of simulating the upstream conditions in the combustion simulations. The upstream turbulent conditions would cause the flame cone to be reduced due to the increased turbulence, thus causing an increase in the combustion rate. However, with the current computational resources simulating the burner inlet nozzle would not be feasible.

**Figure 7:**
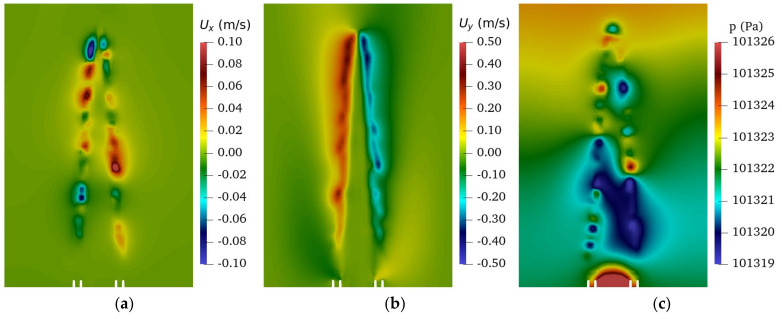
The y–z plane cut of the LES simulation for (**a**) x-component velocity, (**b**) y-component velocity, and (**c**) pressure.

The flame is thought to be a collection of monopole sound sources along the combusting region that is generated in the small turbulent eddies that are produced along the flames edge. A y-z plane cut is plotted in Figure 7 as per the LES simulation. Figure 7a, a contour of the x-component (into the page) velocity, demonstrates this principle with alternating positive and negative quantities, thus illustrating the small turbulent eddies that are produced along the flame edge. In the plot of the y-component (left and right) velocity, Figure 7b, the flow is entrained into the flame region from the surroundings. The pressure, in Figure 7c, also demonstrated the fluctuations being that were by the temperature and turbulence that were generated on the flames edge which directly correlates to the volumetric heat release and sound pressure.

**Figure 8:**
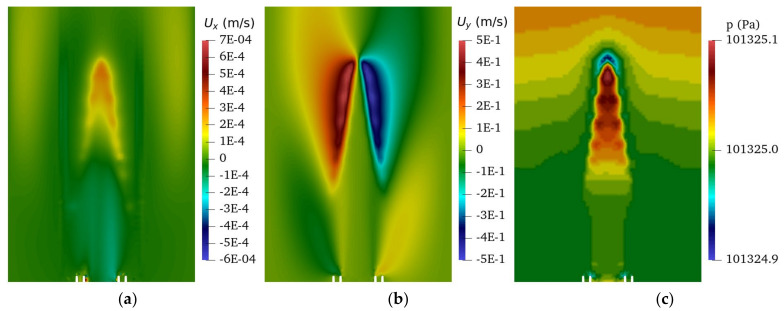
The y–z plane cut of the URANS simulation for (**a**) x-component velocity, (**b**) y-component velocity, and (**c**) pressure.

For comparison, the y-z plane cut was plotted in Figure 8 for the URANS simulation. The contour of the x-component velocity, plotted in Figure 8a, did not show an alternating direction along the flame edge like the LES simulation did. A plot of the y-component velocity, shown in Figure 8b, showed the entrainment region farther from the burner with a reduced magnitude from the LES simulation. The pressure, in Figure 8c, was significantly different; the pressure fluctuation along the edge of the flame is not present in the URANS simulation. Additionally, note that the magnitude of the pressure fluctuations is in the order of 1 Pa in the LES simulation, while it is in the order of 0.1 Pa in the URANS simulation. All of these phenomena can be explained by the averaging of the flow and the resolution of the turbulent scales. The LES was able to resolve the small turbulent scales, thus allowing the appearance of small turbulent eddies to form along the flames edge, while the URANS numerically smoothed these eddies. These turbulent eddies also created the large pressure differences along the flame edge which appeared in the LES, but not in the URANS. These results also provide an explanation for the inaccuracy of the URANS noise results that are presented in the next section.

### 4.2. Noise Results

**Figure 9:**
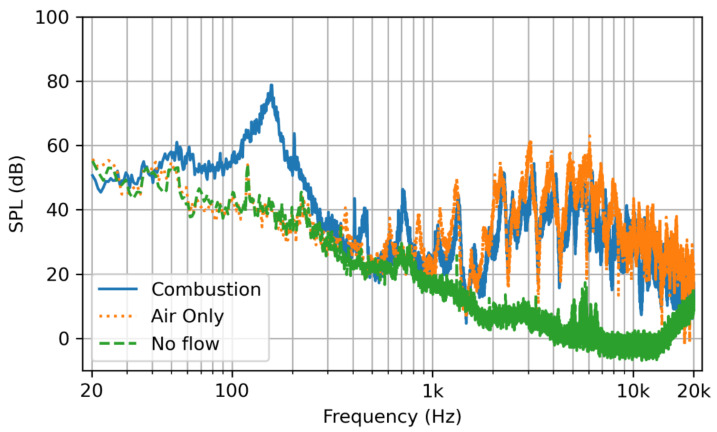
The SPL spectrum for combustion in a solid blue line, air flow only in a dotted orange line, and no flow conditions in the dashed green line.

The SPL spectrum of the combustion noise, air noise, and the ambient noise at the microphone location are plotted in Figure 9. The noise in the lab was significant and may have impacted the results of the simulation. Comparing the noise from the air flow and combustion showed, as expected, that the air flow was the primary contributor to the noise that was of a higher frequency. The upstream flow noise, as well as the noise coming from the piping below the experimental set up likely contributed to this noise and may not be represented appropriately in the simulation. The noise due to combustion was lower frequency, and its peak was around 150 Hz.

**Figure 10:**
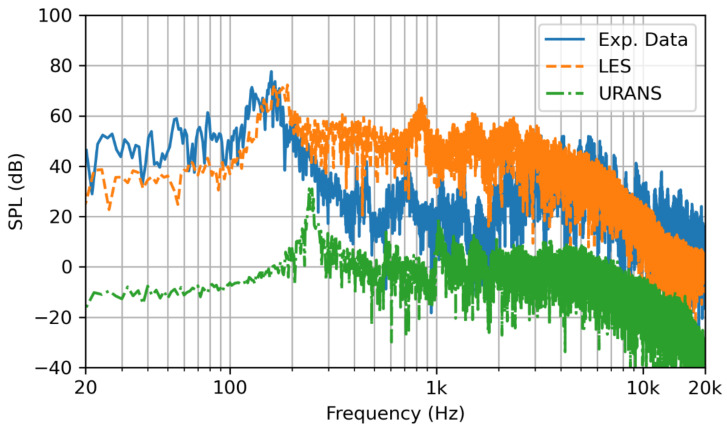
The SPL frequency spectrum for the measured noise (solid blue line) in comparison with the LES modeled noise (orange dashed line) and URANS modeled noise (green dash dotted line).

The SPL frequency spectrum that results from both LES and URANS simulation were compared with experimental data in Figure 10. The LES simulation accurately depicted the peak noise from the combustion. The mid-range frequencies were over predicted. This was attributed to the oscillation in the experiment that were not present in the simulation. These mid-range frequencies were likely a combination of the combustion and the flow, as indicated by Figure 9, which were muffled by the upstream turbulence. Additionally, the large time step contributed to the inaccuracy, thus causing the resolution of the higher frequencies to be diffused. The OASPL of the noise of the LES simulation closely matched the experimental data; 86.7 dB and 86.0 dB, respectively. The URANS-simulated noise amplitude was significantly under-predicted, and the predicted frequencies were significantly higher than they were in the experimental data. The differences between the results from URANS and LES methods are not negligible. For example, in Figure 10, the amplitude and more importantly frequency shift is significant (URANS peak frequency is almost two times of the experimental and LES ones). This difference may be explained partially by the smoothing of the turbulent eddies and the pressure perturbations on the flame front, which is the acoustic source, as demonstrated by comparing Figure 7 and Figure 8.

Interestingly, it was observed that the overall shape of the URANS-simulated noise was similar to the LES-simulated noise. When the frequency and the amplitude of the data were ‘shifted’ by adding 45 dB to the amplitude and subtracting 90 Hz from the frequencies, the URANS data performed similarly to the LES predicted values. More studies are recommended to determine if the offset of the URANS data is predictable over a range of geometries and flow conditions with the goal of producing a scaling law that could be performed post-simulation. As for the results of this study, there were not sufficient data to conclude that the URANS method could be applicable to FWH hybrid method.

## 5. Concluding Remarks

A laboratory scale furnace was built, and the noise from the confined flame was measured using an external sensor. The furnace was simulated using OpenFOAM by the FWH acoustic analogy. The major conclusions are as follows:

The LES/FWH simulation predicted the amplitude and frequency of the primary combustion peak. The LES simulation overpredicted the mid-range frequencies, but the OASPL was in good agreement with the experimental data. An acceptable noise prediction can be achieved with an approximate combustion and flow simulation.

The URANS simulation under-predicted the amplitude of the acoustic noise, and the peak frequencies were shifted higher than those of the experimental data. The differences between the results from URANS and LES methods are not negligible. It was observed, however, that the SPL spectrum was similar in shape, and once it ‘shifted’, it performed similarly to the LES simulation. Future studies should investigate whether a relationship could be determined that would scale the URANS results with post-processing to the levels of the experimental results. If such relationship exists, it could provide a method for implementing an acoustic prediction using the reduced computational requirements.

The externally mounted sensor was successful as a validation tool for the combustion noise of confined flames. Future studies should investigate the effect of the position of the sensor on the experimental results. Additionally, the present results indicate there was ambient noise in the laboratory. While this noise did not seem to reduce the accuracy of the measurements, in future studies, the use of an anechoic chamber and placing the piping and instrumentation in a soundproof box would prevent noise from these sources. 

## Figures and Tables

**Table 1:** Laboratory scale furnace simulation settings.

PIMPLE	nOuterCorrectors	1
	nCorrectors	2
	nNonOrthogonalCorrectors	1
Solvers	rho	diagonal PBiCGStab|DILU PCG|DIC
	U|h|k|epsilon|Yi	
	P	
Numerical Schemes	ddtSchemes	Euler
	gradSchemes	linear
	divSchemes	limitedLinear

**Table 2:** Laboratory-scale furnace boundary conditions.

Boundary	Pressure (Pa)	Temperature (K)	Velocity U (m/s)
Initial Field	uniform 101325	uniform 300	uniform (0 0 0)
outlet	fixedValue	zeroGradient	pressureInletOutletVelocity
inlet	zeroGradient	uniform 300	flowRateInletVelocity
wallPipe	zeroGradient	externalWallHeatFluxTemperature	noSlip
wallBuner	zeroGradient	zeroGradient	noSlip

## Nomenclature

$C_{mix}$	PaSR model mixing coefficient
k	Turbulent kinetic energy
$\vec{r}$	Distance vector
$\dot{V}$	Volumetric flow rate
$y^{+}$	Dimensionless wall distance
$\alpha_{t}$	Thermal diffusivity
ϵ	Turbulent dissipation
λ	Fuel Equivalence Ratio
$\nu_{t}$	Turbulent viscosity
